# Breast implant–associated anaplastic large‐cell lymphoma

**DOI:** 10.1002/ccr3.2135

**Published:** 2019-04-10

**Authors:** Katrina Collins, Joseph A. DiGiuseppe

**Affiliations:** ^1^ Department of Pathology Hartford Hospital Hartford Connecticut

**Keywords:** anaplastic large‐cell lymphoma, breast implant, breast lymphoma, flow cytometry

## Abstract

In patients with suspected breast implant–associated anaplastic large‐cell lymphoma, cytologic evaluation of fine‐needle aspirate specimens from the peri‐implant seroma, together with flow cytometric immunophenotyping and immunohistochemistry, represents a suitable preoperative diagnostic approach when planning for surgical management.

A 50‐year‐old woman with a history of left breast cancer, treated with mastectomy and radiation therapy followed by breast reconstruction with textured silicone‐gel implants five years prior, presented with new‐onset left breast swelling. Microscopic examination of aspirated periprosthetic fluid revealed large, pleomorphic cells (Figure 1A‐D). By flow cytometry (Figure 1E; abnormal cells: blue; normal CD4+ T cells: green; normal CD8+ T cells: red), most of the cells were CD45+ leukocytes with abnormally high side scatter (SSC). The abnormal cells were positive for CD30, and expressed several T‐cell antigens (CD2, CD5, CD7, and CD8; Figure 1E and not shown), but were CD3‐ (Figure 1E). PCR studies demonstrated clonal T‐cell receptor γ‐chain gene rearrangement. A diagnosis of breast implant–associated anaplastic large‐cell lymphoma was made. The subsequently excised periprosthetic capsule (Figure 1F,G) contained large, pleomorphic cells between a layer of eosinophilic material adjacent to the capsular lumen and the underlying capsule. By immunohistochemistry, the neoplastic cells were positive for CD30 (Figure 1H); ALK‐1 was negative (not shown).

**Figure 1:**
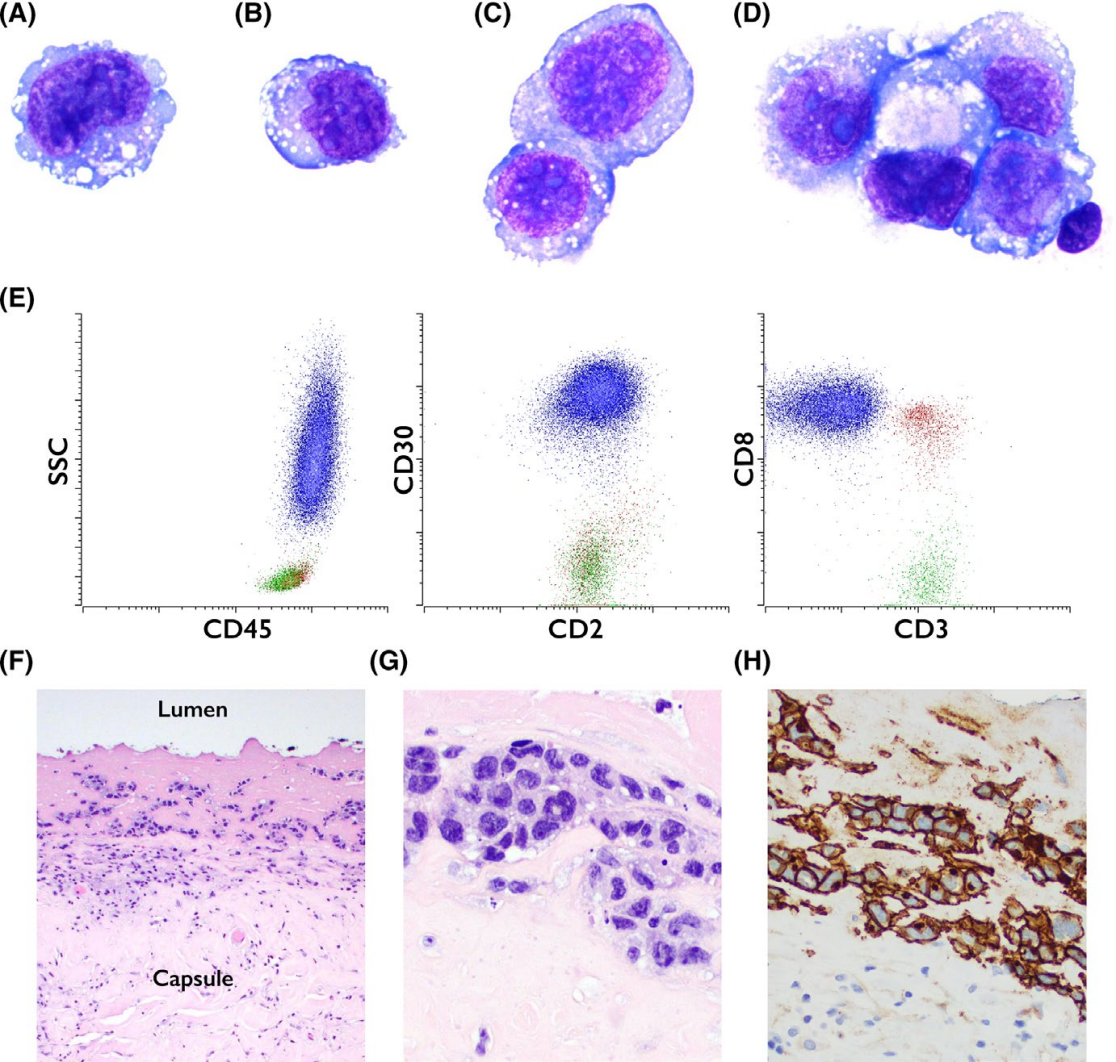
Microscopic examination of aspirated periprosthetic fluid revealed large, pleomorphic cells (A‐D). By flow cytometry (E; abnormal cells: blue; normal CD4+ T cells: green; normal CD8+ T cells: red), most of the cells were CD45+ leukocytes with abnormally high side scatter (SSC). The abnormal cells were positive for CD30, and expressed several T‐cell antigens (CD2, CD5, CD7, and CD8; E and not shown), but were CD3‐ (E). The subsequently excised periprosthetic capsule (F,G) contained large, pleomorphic cells between a layer of eosinophilic material adjacent to the capsular lumen and the underlying capsule. By immunohistochemistry, the neoplastic cells were positive for CD30 (H); ALK‐1 was negative (not shown)

Breast implant–associated anaplastic large‐cell lymphoma (BIA‐ALCL) is a recently recognized provisional diagnostic entity in the Revised 4th Edition of the WHO classification of lymphoid neoplasms.^1^ This rare form of T‐cell non‐Hodgkin lymphoma, which appears to be related to textured implants, arises after a highly variable latency that averages approximately 10 years.^2^, ^3^ Patients most commonly present with a collection of fluid around the implant (seroma), often associated with swelling, pain, asymmetry, or mass lesion in the breast or armpit.^4^, ^5^ Although optimal management has not yet been firmly established, complete surgical excision of the periprosthetic capsule with implant removal is considered important.^5^ Preoperative diagnosis of BIA‐ALCL is therefore helpful in planning surgical management. Because the neoplastic cells are commonly suspended within the seroma fluid, cytologic evaluation of fine‐needle aspirate specimens, together with flow cytometric immunophenotyping^6^, ^7^ and immunohistochemistry, represents a suitable preoperative diagnostic approach, as illustrated in the current case.

This work was presented in preliminary form at the College of American Pathologists 2018 Annual Meeting (CAP18).^8^

## CONFLICT OF INTEREST

None declared.

## AUTHOR CONTRIBUTION

KC and JD: contributed to the design and implementation of the research, analysis of the results, and writing of the manuscript.
